# Identification and molecular characterization of *Brucella abortus* and *Brucella melitensis* isolated from milk in cattle in Azerbaijan

**DOI:** 10.1186/s12917-022-03155-1

**Published:** 2022-02-15

**Authors:** Jeyhun Aliyev, Mahnur Alakbarova, Aytan Garayusifova, Asaf Omarov, Saida Aliyeva, David Fretin, Jacques Godfroid

**Affiliations:** 1 Department of Epizootology, Microbiology and Parasitology, Faculty of Veterinary Medicine, ASAU – Azerbaijan State Agricultural University, 450, Ataturk Avenue, AZ 2000 Ganja, Azerbaijan; 2 Goygol Regional Testing Laboratory, Azerbaijan Food Safety Institute, Heydar Aliyev Ave. 142, Goygol, Azerbaijan; 3 Department of Epidemiology, School of Public Health, UW – The University of Washington, 1959 NE Pacific St, Seattle, WA USA; 4 Life Science Department, Khazar University, 41 Mehseti Street, AZ1096 Baku, Azerbaijan; 5 Black & Veatch Special Projects Corp, Maryam Plaza, 12 B.Baghirova, AZ1065 Baku, Azerbaijan; 6 Sciensano, Veterinary Bacteriology, Groeselenberg 99, 1180 Brussels, Belgium; 7 Department of Arctic and Marine Biology, Faculty of Biosciences, Fisheries and Economics, UiT - The Arctic University of Norway, Postbox 6050, Langnes, 9037 Tromsø, Norway

## Abstract

**Background:**

Azerbaijan currently ranks thirteenth in global incidence of human brucellosis, with an estimated annual incidence through 2000 at over 50 cases per million. *Brucella melitensis* has been isolated from patients and is thought to have been acquired through contact with small ruminants or as a foodborne infection. To reduce the burden of human brucellosis, the Azerbaijani government began in 2002, a nationwide vaccination control campaign in small ruminants. There is serological evidence of bovine brucellosis (presumably due to *Brucella abortus*) in Azerbaijan, but no prevalence estimates were available when this study started in March 2017. The aim of this study was to isolate and identify *Brucella* spp. from cow milk in the Ganja region, where brucellosis takes a heavy toll on humans and livestock.

**Results:**

Blood and milk samples were collected from cows (*n* = 1075) in early lactation (up to 90-days) in farms that had a history of previous positive serological results and abortions. Twenty-two out of 57 milk samples collected from seropositive cows, showed growth on Farrell’s media, when incubated with 5% CO_2_. Eight additional milk samples showed growth in the absence of CO_2_. The classical biotyping classified them as *Brucella abortus* (22) and *Brucella melitensis* (8). RT-PCR confirmed that strains belonged to the genus *Brucella*. MLVA profiles were obtained for DNA extracted from two *B. abortus* and six *B. melitensis* strains. While the *B. abortus* genetic profile was described in the MLVA database, matching the profile of *B. abortus* strains isolated in East Europe, Central Asia and China, we found a new genotype for the *B. melitensis* strains isolated in Azerbaijan, clustering with strains belonging to the American clade, rarely identified in the region.

**Conclusion:**

Despite the implementation of the vaccination program in small ruminants, our results suggest that spill-over events of *B. melitensis* from small ruminants to cattle have occurred. However, cattle are likely to be primarily infected with *B. abortus,* which warranted the implementation of a bovine brucellosis program. Such a program started in fall 2017. In the Ganja region, cattle should be considered as a potential source of *B. abortus* and *B. melitensis* for humans.

**Supplementary Information:**

The online version contains supplementary material available at 10.1186/s12917-022-03155-1.

## Background

Brucellosis is the most common bacterial zoonosis globally, causing a debilitating human disease [1, 2]. Human brucellosis is reported worldwide, with at least 500,000 people newly infected annually [3]. Human cases of brucellosis are acquired through contact with infected animals and consumption of contaminated dairy products [3]. The decrease in the incidence of human cases depends on developing effective control and eradication programs in cattle and small ruminants [4, 5]. Among the *Brucella* species that infect animals, five are known to cause disease in humans, with *Brucella abortus, Brucella melitensis and Brucella suis* being the most important in terms of clinical severity and prevalence [6]. *Brucella melitensis* is considered the most prevalent and virulent *Brucella* species for humans [1]. The human disease typically presents an acute, nonspecific illness characterized by undulating fever, malaise, myalgia, and weight loss that may resemble other acute febrile diseases. A chronic form of brucellosis lasting longer than 12 months after diagnosis can also occur [2, 7]. *Brucella* infection in women can cause abortions or labor complications and in men, orchitis and epididymitis [7].

Brucellosis in humans was identified in Azerbaijan in 1922 for the first time and was subsequently detected in more than two-thirds of the country’s districts over the next 30 years [8]. Of the 62 countries identified as having the highest national incidence of human brucellosis, Azerbaijan currently ranks thirteenth, with an estimated annual human incidence through 2000 at over 50 cases per million [2, 9, 10]. According to a press release issued by the Ministry of Health of Azerbaijan (MoH) in August 2014, approximately 200 human cases of brucellosis were diagnosed in the first 6 months of that year; this detection rate was similar to what was reported in the previous year [11].

*Brucella infection in livestock causes* significant economic losses through the reduction of productivity. The acute form of the disease are characterized by abortion in pregnant females and orchitis and epididymitis in males, whereas the chronic forms is characterized by hygromas, mainly seen in the tarso-metacarpal joints, inducing lameness in infected animals [12]. The main *Brucella* species isolated from livestock worldwide are *B. abortus,* infecting preferentially bovines; *B. melitensis,* infecting preferentially sheep and goats; and *B. suis*, infecting preferentially pigs [2, 13]. International veterinary regulations impose restrictions on animal movements and trade in countries that are not “Brucellosis-free,” resulting in substantial economic costs due to the implementation of control and eradication programs in cattle and small ruminants worldwide [14].

The collapse of the Soviet Union in 1991 resulted in decreased funding for surveillance and eradication programs in livestock and an increase in human cases. To reduce the burden of human and livestock brucellosis, the Azerbaijani government decided in 2002, to implement a nationwide vaccination control campaign in small ruminants.

Small ruminants are vaccinated against *B. melitensis* with the Rev. 1 vaccine [15]. Female sheep and goats between the ages of 3 and 6 months are vaccinated intra-conjunctively, before being moved to summer pasture. Male animals are not vaccinated. In 2016, 6,400,000 heads of small ruminants were vaccinated [16]. At the time our study, cattle were not vaccinated against *Brucella* spp. [17]. The vaccination with the S-19 vaccine of 3 to 8 month-old cattle started in 2017 [16].

Livestock population estimates for 2020 were about 2.6 million cattle and buffalo, which about 1.2 million were dairy cows and about 77 thousand were dairy buffalo. The small ruminant population comprised about 8 million sheep and about 613,800 goats. There are only about 5700 swine. Livestock numbers have been relatively stable since 2010 [18].

Previous brucellosis studies conducted in Azerbaijan were mainly serological surveys and published information is scarce in the scientific literature. Based on the World Organization for Animal Health (OIE) data for Azerbaijan, brucellosis in cattle, sheep and goats was not reported from 2005 to 2007. Since 2008, data has been annually reported [15]. For instance, 400 - 500 humans, 1100 cattle; and 1200 sheep and goats were found seropositive for brucellosis in Azerbaijan in 2011 [18, 19]. There were 2230 brucellosis cases registered in Azerbaijan in 2017–2018 [15, 16].

When cattle are in contact with infected small ruminants, *B. melitensis* can infect bovines too [20]. Identifying the species of *Brucella* excreted in milk of dairy cattle is needed to understand the transmission of *Brucella* spp. within and between cattle herds, as well as between livestock species in mixed (bovines/small ruminants) herds. Importantly, it is also needed to trace back the source of infection of human brucellosis. In case of human infection with *B. melitensis*, the source of contamination can be either small ruminants or bovines. Importantly, the identification of *Brucella* species in non-preferential hosts (like *B. melitensis* in bovines), allows to assess if control measures applied in small ruminants are successfully implemented and prevent the transmission of *B. melitensis* from its small ruminant reservoir to cattle.

This study aimed to identify and characterize *Brucella* species circulating in lactating cattle in brucellosis high-risk villages in the districts serviced by the Goygol Testing Laboratory (RTL). The Goygol district was selected as the site of this study for several reasons, including the extent of livestock production, mixed herding (cattle/sheep and goat) and use of communal pastures, and the known presence of brucellosis in livestock and humans [21]. Moreover, transhumance is also common practice and the migratory animal population may be at the origin of new infections in non-migratory livestock in the Goygol district.

## Results

### Sample collection

A total of 1075 serum and milk samples were collected from 368 farms between April and August of 2017. Two hundred thirty-three milk and serum samples were collected from Goygol; 120 milk and serum samples were collected from Dashkesen; 160 milk and serum samples were collected from Ganja; 360 milk and serum samples were collected from Goranboy, and 202 serum milk samples were collected from Samukh.

### Laboratory testing

Fifty-seven lactating cows were classified positive by the Rose Bengal Test (RBT). Milk samples from these seropositive animals were cultured on BA with the following results: 54 out of 57 cream samples were culture positive, while 44 out of 57 pellet samples were culture positive. For each milk sample from which the pellet sample was culture positive, the cream sample was also positive. Eight out of 57 milk samples showed growth on BA incubated in a standard incubator, without CO_2_. These eight isolates showed growth on both Basic Fuschin and Thionin and did not produce H_2_S. For these eight isolates, in the absence of phage typing, a presumptive diagnosis of *B. melitensis* was obtained. Of note, *B. abortus* biovar 6 (that has never been isolated in the region) cannot be differentiated from *B. melitensis*, based on the phenotypic tests performed [22]. Thirty (including eight samples that showed growth in the absence of CO_2_) out of 57 milk samples showed growth on BA incubated with 5% CO_2_. All isolates were oxidase, catalase, urease positive. Twenty-two isolates that showed 5% CO_2_ dependence showed growth on Basic Fuchsin, no growth on Thionin, and produced H_2_S. A presumptive diagnosis of *B. abortus* was obtained.

**Fig. 1:**
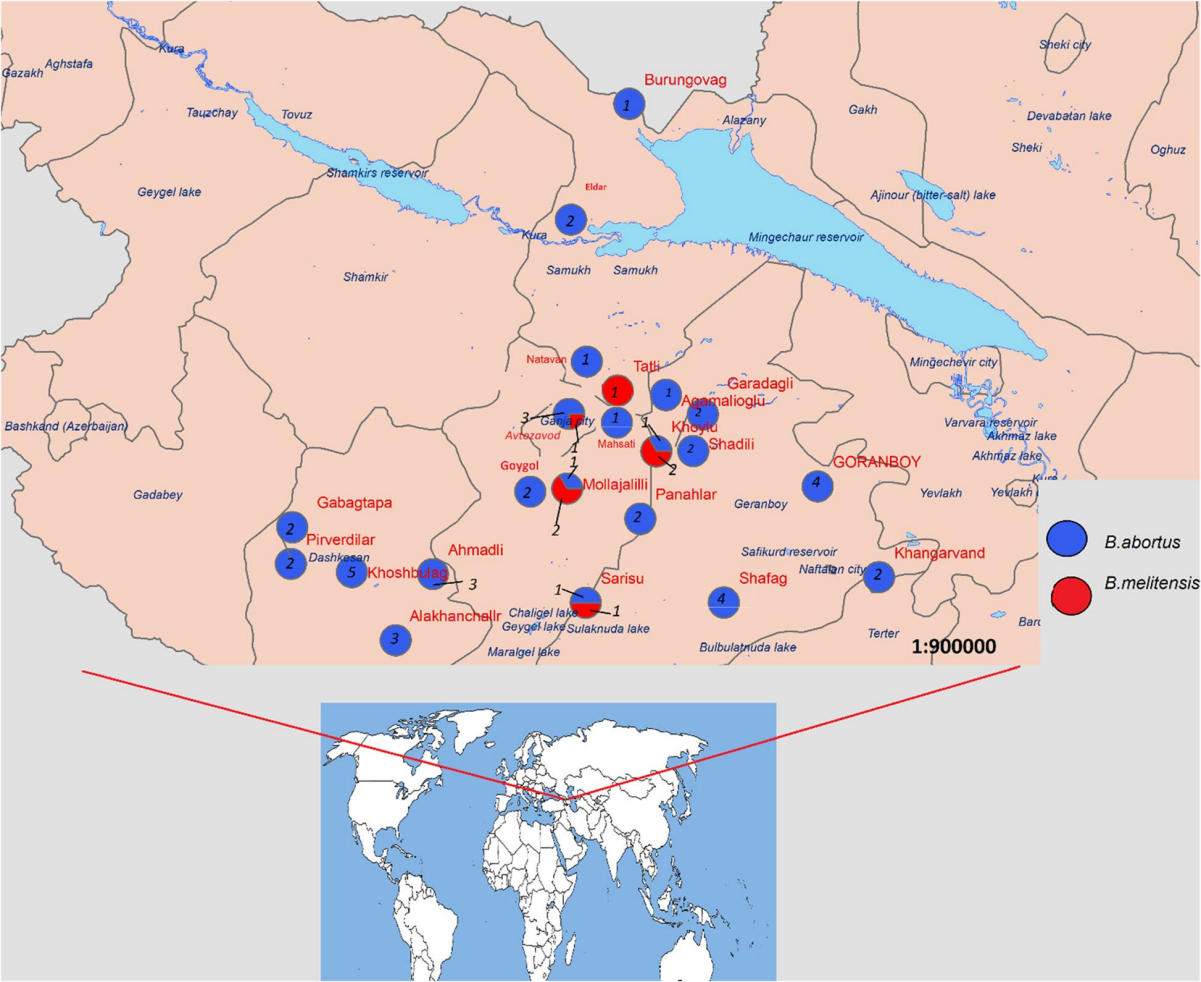
Location where *B. abortus* and *B. melitensis* were isolated. The number in the circle indicates the number of isolated strains from the same location Both *B. melitensis* and *B.abortus* were found in 5 villages (*Mollajalilli, Sarisu, Avtozavod, Shadli, Khoylu*). Only *B. abortus* was found in 17 villages (Goygol, Panahlar, Eldar, Brungovag, Mehseti, Natavan, Sadili, Khoshbulag, Qabaqtepe, Pirverdi, Alkhanchali Shafag, Agamalioglu, Khan Garvand, Garadagli) while *B. melitensis* only was found in one village (Tatli)). 3 *Brucella* spp. were isolated from Mollajalilli out of 3, 2 were *B. melitensis,* 1 was *B.abortus, 2 Brucella* spp. were isolated from Sarisu 1 was *B.melitensis,* 1 was *B. abortus.* 4 *Brucella* spp. were isolated from Avtozavod. 3 were *B. abortus,* 1 was *B. melitensis, 5 Brucella* spp. were isolated from Shadili. Out of 5, 1 was *B. melitensis,* 4 were *B. abortus,* 3 *Brucella* spp. were isolated from Khoylu*.Out of 3,* 2 were *B. abortus,* 1 was *B. melitensis.* The figure was created using ArcGIS 10.3 (https://desktop.arcgis.com/en/arcmap/10.3/get-started/installation-guide/installing-on-your-computer.htm)

All of the isolates were Gram-negative coccobacilli and tested positive by RT-PCR for *Brucella* spp. Four out of 30 *Brucella* spp. infected cows had previously aborted, 14 had contact with sheep, 26 had natural and 2 artificial insemination, and 2 were first lactating heifers. *Brucella melitensis* was isolated from cattle in Goygol, Samukh, and Goranboy districts and Ganja city, while *B. abortus* was found in all five locations surveyed. A positive sample distribution map is presented in Fig. 1.

### Multiple-locus variable number tandem repeat analysis (MLVA) results

Six isolates growing in the absence of CO_2_ and two isolates growing exclusively in the presence of CO_2_ were characterized by MLVA as *B. melitensis* and *B. abortus*, respectively.

**Fig. 2:**
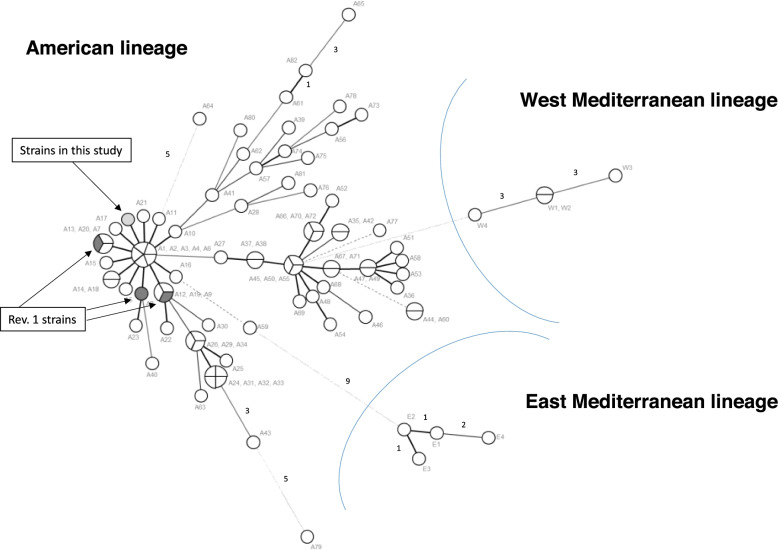
Minimum Spanning Tree with MLVA-16 data of the *B. melitensis* isolated in Azerbaijan. Strains isolated from cattle milk (light grey) compared with *B. melitensis* strains belonging to the American lineage, including Rev. 1 strains (dark grey). and to a selection of *B. melitensis* genotypes belonging to the West and East European lineages (MLVA profiles derived from publicly available data (http://mlva.u-psud.fr/) and labelled as in Additional file 1. Clustering by minimum spanning tree was performed with Bionumerics version 6.6 software (Applied Maths, Sint-Maartens Latem, Belgium). Circles outline the genetic profiles of strains. Numbers on the connecting lines refer to the number of markers differing between samples. The size of the circles is proportional to the number of strains (1-4) bearing the same genetic profile

The six *B. melitensis* strains showed the same genetic profile (3-4-2-13-4-2-3-3-8-36-6-1-4-6-3-6) that was not reported in the MLVA 2020 database. This new genotype is clustering with the genotype of strains belonging to the “Americas” lineage (Fig. 2 and Additional file 1) [23, 24].

When compared to the *Brucella* MLVA 2020 database, the *B. abortus* strains showed the same genetic profile (4-5-3-12-2-2-3-1-6-43-8-6-4-5-3-3), reported previously (Additional file 2). On the global scale, MLVA-16 assay reveals little diversity of *B. abortus* and suggests an Eurasian distribution of this lineage found in China, Turkey, and Kazakhstan [25].

## Discussion

By carefully selecting animals to be sampled, on the bases of previous epidemiological information and RBT testing, we were able to culture *Brucella* spp. from cow milk. Standard microbiological biotyping (OIE, 2016) and MLVA confirmed that both *B. abortus* and *B. melitensis* were circulating in the cattle population in Azerbaijan.

Globally, *Brucella melitensis* strains cluster into three main distinct MLVA lineages: the “American”, “West Mediterranean,” and “East Mediterranean” lineages [23]. Multilocus sequence analysis (MLST) of *B. melitensis* strains has confirmed the MLVA classification [26]. In 2018, an additional African lineage of *B. melitensis* was identified [27]. The “American” lineage is also named the “Americas group” or “American clade”. It should be emphasized that the American clade, like the African clade are phylogenetic assignments and not geographic ones [27]. Of note, more than 60% of the isolates of the American clade are of African origin [26].

The *B. melitensis* strains isolated in Azerbaijan from cattle milk belong to the American lineage. The Rev. 1 vaccine strain belongs to this lineage too [27]. Given the extensive use of Rev.1 in small ruminants in Azerbaijan, it is important to verify that *B. melitensis* strains isolated in cattle are not Rev.1 strains transmitted by vaccinated sheep and goats. The classical biotyping of the six *B. melitensis* strains showed that all strains grew in the presence of Thionin and Basic Fuchsin. Rev.1 does not grow in presence of these dyes. We can therefore exclude that these strains are Rev.1 strains or have evolved from the vaccine strains.

*Brucella melitensis* strains belonging to the American clade have been previously reported in Asia. Indeed, such strains have been isolated from blue sheep “Bharal” (*Pseudois nayaur*) in China, typed by MLVA [28] and from human patients in Afghanistan, Syria, Saudi Arabia and Iran, typed by MLST [27].

The MLVA and MLST genotypes of *B. abortus* show a greater homogeneity than *B. melitensis* [26]. The MLVA of the two *B. abortus* strains isolated from cow milk in this study were identical to the MLVA of *B. abortus* strains isolated in China, Turkey, and Kazakhstan [25].

Although brucellosis endemic to Azerbaijan, only a handful of *Brucella* bacteria have been isolated from human patients. All of these clinical isolates were identified as *B. melitensis* strains [29]. This suggested that the main reservoir for human transmission was small ruminants. This has been the prevailing assumption in Central Asia and Eastern Europe [29]. Our study shows that *B. melitensis* can be shed in cattle milk. This suggests that cattle can be the source of human brucellosis due to *B. melitensis*, as it has been shown in France [20]. To date, the isolation of *B. abortus* from human patients has not been reported in Azerbaijan. Nevertheless, our study indicates that *B. abortus* is shed in cow milk and suggests that humans may be exposed after consumption of cattle milk. When this study was conducted, there was no vaccination program in cattle in Azerbaijan, whereas vaccination of small ruminants with Rev. 1 has been ongoing since 2016, when over 6,400,000 heads of sheep and goats were vaccinated [16]. The fact that 6 out of 30 culture-positive cows were excreting *B. melitensis* in their milk, suggests that there are still some foci of *B. melitensis* infection in small ruminants in which transmission to cattle may have occurred. Therefore, a critical evaluation of the Rev.1 vaccination program in small ruminants should be performed. To this effect, antibody titers of vaccinated animals can inform vaccination coverage and efficacy. Studies in Mongolia suggested that 60% of the small ruminant population should be vaccinated to reach a protective “herd immunity” [30].

Because 14/30 *Brucella* positive cows were found to be infected with *B. melitensis,* we suggest that *B. melitensis* infected small ruminants remain an important source of cattle infection. To confirm our hypothesis, *B. melitensis* strains should be isolated from sheep and goats for genetic comparison. Cows excreting *B. melitensis* may further transmit the infection to other bovines and humans. Importantly, the majority of the strains isolated from cow milk are *B. abortus*. Although *B. abortus* has not been isolated from human patients, a vaccination program of cattle in Azerbaijan in high-risk areas has been started in 2017.

Our results suggest that the current vaccination campaign in small ruminants should be monitored by performing serology on animals a month after vaccination to assess vaccination coverage and vaccine efficacy. In addition, isolation of *B. abortus* raised the question of whether vaccination against *B. abortus* infection in cattle should be considered.

## Conclusion

This study highlights the importance of strain isolation, identification, and characterization to understand the epidemiology of brucellosis in an endemic situation. Indeed, the isolation of *B. melitensis* in a significant proportion (> 20%) out of *Brucella* spp*.* excreted in cow milk, suggests that the vaccination campaign against *B. melitensis* in small ruminants has not yet controlled *B. melitensis* circulation in its reservoir species so that *B. melitensis* has spilled over to cattle. Besides, the isolation of *B. abortus* in a large proportion (> 70%) out of *Brucella* spp*.* excreted in cow milk suggests that vaccination of cattle may be warranted.

The isolation of *B. melitensis* from cow milk suggest that strains are circulating between sheep, goats, and cattle in Azerbaijan. The isolation and typing of strains isolated from sheep and goats are needed to establish such epidemiological links. Our MLVA results show that the isolated strains belong to the American clade that is not the dominant clade of *B. melitensis* strains circulating in the region. Indeed, only a handful of strains belonging to the American clade have been isolated in blue sheep in China. Importantly, strains belonging to this clade have been isolated from human patients in the Middle East and Asia. Thus, it suggests that the reservoirs of such strains in livestock may be larger than thought. Indeed, no isolation of strains belonging to the American clade has been reported in neighboring countries, either in sheep and goats or cattle.

The typing of *B. abortus* isolated from cattle milk suggests that strains circulating in Azerbaijan belong to the same clade as strains isolated from East Mediterranean countries to China, suggesting a circulation of strains linked to animal transhumance and trade among these countries.

The vaccination program in sheep and goats needs to be continued in order to stop transmission between sheep and goats, and cattle in Azerbaijan.

The isolation of *B. abortus* provides the Veterinary Authority in the Country with an evidence base information validating its decision to implement S19 vaccination in cattle, with 398,306 animals (3-8 months of age) being vaccinated in 2017.

Lastly, new techniques such as Whole Genome Sequencing (WGS) applied on strains isolated in the region in different livestock species will help to decipher the epidemiology of animal brucellosis and its transmission to human patients in the future [31].

## Methods

### Field activity

The study was performed in Goygol, Dashkesen, Samukh, Goranboy, and Ganja city. Before the 2017 sample collection, the data on seropositive animals and *Brucella* high-risk farms in those regions were extracted from the Electronic Integrated Diseases Surveillance System (EIDSS) database [16]. Lactating cows that were not treated with antibiotics during the lactation period were selected for this study. The investigators collected blood and milk samples from cows in early lactation (up to 90-days) in farms that had a history of previous positive serological results and abortions. In addition, lactating first-calf heifers that were likely exposed to *Brucella* spp., were also sampled. Approximately 5 mL of blood was collected into serum-separator Vacutainer® (Becton Dickinson, NJ, USA) and 50 mL of milk into a sterile conical tube. The blood and milk samples were transported to RTL on the day of sample collection.

### Serology

Serum samples were screened using the RBT (Oxoid, Basingstoke, Hants, UK), according to OIE [12].

### Bacteriology

Milk samples that were collected from RBT positive animals were cultured. Milk samples were centrifuged at 3000 rpm for 15 min at 4 °C. Cream and pellet samples were cultured separately on *Brucella* agar base no. 2 with Oxoid *Brucella* selective supplement (Thermo Fisher Scientific, UK), 7% horse serum, and 1% glucose and incubated in a standard (without CO_2_) and in a microaerobic (5% CO_2_) chamber. Colonies were identified as *Brucella* spp. through classical identification methods, according to OIE [22].

### PCR detection

DNA was extracted from bacteria grown on *Brucella* selective media using Qiagen QIAmp DNA Mini (Qiagen, Hilden, Germany). RT-PCR was performed to test for the presence of *Brucella* spp. using primers and TaqMan probes utilized for the simplex assay (Qiagen, California, USA) as described previously [32]. The quantity of DNA was not assessed.

### Multiple-locus variable number tandem repeat analysis (MLVA)

Genotyping was performed using both minisatellites and microsatellite repeats based on the schemes previously described [23, 33]. The tandem-repeat loci were divided into three groups: four minisatellite loci in panel 1 (bruce08, bruce12, bruce43, bruce45), two microsatellite loci in panel 2A (bruce18, bruce19) [34].

For the markers bruce08, bruce12, bruce43, bruce45, bruce18, bruce19, the PCR product length was defined by capillary electrophoresis with the CEQ 8000 Genetic Analysis System (Beckman Coulter, Indianapolis, IN, USA). The size of each PCR product was then converted to a corresponding tandem repeat number [33]. All data were analyzed using BioNumerics version 6.6 software (Applied Maths, Sint-Maartens Latem, Belgium).

Clustering analysis was performed using the categorical coefficient and the unweighted-pair group method with arithmetic mean algorithm (UPGMA) as indicated previously [34]. Briefly, three distinct character data sets with different weights were defined according to the markers’ diversity index and combined using the composite data set tool provided by BioNumerics. The first one corresponded to panel 1 markers. Each marker of this panel got an individual weight of 2 (total weight for panel 1: 16). The two others form two groups in panel 2, 2A and 2B. Panel 2A markers got a weight of 1 (total weight for panel 2A: 3) and panel 2B markers got a weight of 0.2 (total weight of panel 2B: 1). The MLVA profile of the isolates was also subjected to a minimum spanning tree (MST) analysis, illustrating the diversity existing within the clusters based on single locus variations (SLV). Units (and not sizes) of each marker were considered for the analysis [35].

GPS data was collected during sampling process and ArcGIS 9 (Environmental systems research institute, Redlands, CA, USA) was used to generate the map (Fig. 1).

## Supplementary Information

**Additional file 1. **MLVA *Brucella melitensis* lineages 2020 database.

**Additional file 2. **MLVA *Brucella abortus* isolated in China, Turkey, Kazakhstan.

## Data Availability

The datasets analyzed during the current study are available from the corresponding author on reasonable request.
